# Targeting Synaptic Plasticity in Experimental Models of Alzheimer’s Disease

**DOI:** 10.3389/fphar.2019.00778

**Published:** 2019-07-16

**Authors:** Dalila Mango, Amira Saidi, Giusy Ylenia Cisale, Marco Feligioni, Massimo Corbo, Robert Nisticò

**Affiliations:** ^1^Laboratory of Neuropharmacology, EBRI Rita Levi-Montalcini Foundation, Rome, Italy; ^2^Department of Physiology and Pharmacology, Sapienza University of Rome, Italy; ^3^Department of Neurorehabilitation Sciences, Casa Cura Policlinico, Milan, Italy; ^4^School of Pharmacy, Department of Biology, University of Rome Tor Vergata, Rome, Italy

**Keywords:** long-term potentiation, long-term depression, synaptic plasticity, Alzheimer’s disease, predictive validity

## Abstract

Long-term potentiation (LTP) and long-term depression (LTD) of hippocampal synaptic transmission represent the principal experimental models underlying learning and memory. Alterations of synaptic plasticity are observed in several neurodegenerative disorders, including Alzheimer’s disease (AD). Indeed, synaptic dysfunction is an early event in AD, making it an attractive therapeutic target for pharmaceutical intervention. To date, intensive investigations have characterized hippocampal synaptic transmission, LTP, and LTD in *in vitro* and in murine models of AD. In this review, we describe the synaptic alterations across the main AD models generated so far. We then examine the clinical perspective of LTP/LTD studies and discuss the limitations of non-clinical models and how to improve their predictive validity in the drug discovery process.

## Introduction

Long-term synaptic plasticity is considered the neural basis of learning and memory process (Bliss and Collingridge, 1993). Long-term potentiation (LTP) and long-term depression (LTD) are the major forms of durable synaptic strength changes in central nervous system abundantly studied in the hippocampal region (Malenka and Bear, 2004). The magnitude of LTP and LTD is largely used in many different experimental conditions and animal models as an indicator of cognitive function; on the other hand, dysregulation of synaptic plasticity underlies a large number of neurodegenerative disorders such as Alzheimer’s disease (AD) (Selkoe, 2002).

AD is a multifaceted neurodegenerative disorder typified by a progressive and irreversible memory deficits and cognitive decline. To date, AD can only be diagnosed *post-mortem*, through two characteristic neuropathological lesions in the brain: senile plaques, consisting of β-amyloid protein oligomers aggregates (Aβo, residues 1–40/42), and intracellular neurofibrillary tangles (NFT), constituted of abnormally hyperphosphorylated tau protein accumulation predominantly in hippocampal and cortical regions. The “amyloid cascade hypothesis” is so far the prominent theory to describe the time-course of AD neurodegeneration (Hardy and Higgins, 1992). Impaired synaptic function of the hippocampus is an early event leading to defective hippocampal-dependent memory appearing long before the buildup of amyloid plaques and neuronal cell death (Selkoe, 2002; Tanzi, 2005). Therefore, synaptic plasticity is often used to evaluate part of the phenotype. Accordingly, many electrophysiological studies on the different models have been performed to delineate such changes.

Early impairments in synaptic transmission were highlighted in different mouse models of AD and are caused, among other factors, by Aβ which leads to impairment of LTP *via* tau protein (Shipton et al., 2011). Notably, many studies investigated the correlation between age and synaptic dysfunction in order to describe the onset and development of pathology in a specific mouse model. Discrepancy in the results obtained by the different researchers across the various models of AD may reflect the type of mutation studied, in addition to several other sources of variations such as experimental design, age, or strain.

## LTP and LTD in Normal Conditions

Several studies indicate that the hippocampus plays a crucial role in higher cognitive functions and in information-storage (reviewed by Neves et al., 2008). LTP was first studied in the hippocampus and has been widely characterized using biochemical, electrophysiological, and molecular techniques (reviewed by Bliss et al., 2007). LTP is characterized by a short-term phase (early- or E-LTP) and a subsequent long-term phase of potentiation (late- or L-LTP) (Reymann et al., 1989). Importantly, distinct forms of N-methyl-D-aspartate (NMDA) receptor LTP coexist at synapses (Park et al., 2014) and these can also be distinguished based on their responsiveness to protein kinase A (PKA) inhibitors (Park et al., 2016). E-LTP and L-LTP can be induced in hippocampal slices by different induction protocols and are sustained by distinct cellular and molecular pathways. E-LTP (<1 h) is characterized by the recruitment of postsynaptic 2-amino-3-(3-hydroxy-5-methyl-isoxazol-4-yl)propionic acid (AMPA) receptors, either from neighboring extra synaptic receptors or from intracellular reserve pool by exocytosis (Penn et al., 2017). On the other hand, L-LTP (>3 h) involves *de novo* protein synthesis promoting structural and functional changes (Frey et al., 1988). Among the key players facilitating the transition from E-LTP to L-LTP, brain-derived neurotrophic factor (BDNF) (Panja and Bramham, 2014) and transforming growth factor β1 (TGF-β1) (Caraci et al., 2015; Caraci et al., 2018) are noteworthy.

The classical form of LTD can be experimentally elicited using specific electrical low-frequency stimulation (LFS) protocols in slices (Dudek and Friedlander, 1996). Most of the LTD forms studied imply activation of NMDA receptors (Dudek and Friedlander, 1996) and/or metabotropic glutamate (mGlu) receptors (Fitzjohn et al., 1999). Other chemical forms of LTD are obtained either by exogenous application of NMDA or muscarinic receptor agonists (reviewed by Collingridge et al., 2010), as well as through activation of microglia (Zhang et al., 2014).

## LTP and LTD in Experimental AD

### *In Vitro* Models

The peptide amyloid beta is released endogenously during physiologic neuronal activity and causes enhancement of synaptic plasticity and memory formation when administered at picomolar concentrations that likely resemble the physiological level in the brain (Puzzo et al., 2008; Morley et al., 2010; Puzzo et al., 2011; Lawrence et al., 2014). Conversely, a prolonged exposure to the same amount is able to impair synaptic plasticity by glutamate-induced excitotoxicity (Koppensteiner et al., 2016). Specifically, it has been demonstrated that NR2B-containing NMDA receptors and mGlu5 receptors mediate the synaptotoxic effects of Aβo (Rammes et al., 2017).

The effects of acute application of exogenous Aβ oligomers (Aβo) obtained from synthetic, secreted from AD transgenic cells, or extracted from AD patients’ brain, on synaptic transmission, have been widely studied in *ex vivo* hippocampal slices. All studies suggest that treatment of hippocampal slices with Aβo 200–500 nM induces alteration in LTP and LTD, generally manifested as loss of LTP and enhancement of LTD (Lambert et al., 1998; Wang et al., 2002; Shankar et al., 2008; Li et al., 2009; Jo et al., 2011; Cavallucci et al., 2013; Mango et al., 2016). Additionally, another study has shown that over-expression of Aβ in organotypic slices reduces the number of surface L-α-amino-3-hydroxy-5-methyl-4-isoxazole propionate receptor (AMPAR) similar to what occurs in mGlu receptor-dependent LTD (Kamenetz et al., 2003). Recently, we have demostrated that hippocampal mouse slices treated with Aβo display an enhancement of mGlu receptor-dependent LTD (Mango and Nisticò, 2018).

### AD Mouse Models

Animal models of AD should fully model human features of disease including gradual cognitive decline, synaptic dysregulation and spine loss, plaque load and NFT accumulation, inflammation, neurodegeneration, and atrophy of the central nervous sistem (CNS).

The breakthrough of amyloid precursor protein (APP) and PS human mutations has led to the generation of transgenic (Tg) animal models which strictly replicate the cardinal features of AD. AD models are largely used to explore in a spatiotemporal manner the pathogenic mechanisms of AD and the benefit of therapeutic approaches.

Several lines of transgenic models of AD have been generated so far; each recapitulates specific aspects of the disease. They exemplify more integrated approaches to examine the complex effects of Aβo on brain integrity and network function. Several mouse models have been so far analyzed for hippocampal synaptic function by means of electrophysiological techniques (see **Table 1**). To simplify, we divide them in APP-derived, PS1-derived, APP/PS1, 3xTg, and 5xTg models. Most of these models are constructed on the overexpression of familial AD (FAD)-linked mutated genes into the mouse genome, and magnitude of LTP and LTD has been used to study synaptic plasticity alterations. Several exhaustive reviews on this topic have been published in the literature (Morrissette et al., 2009; Ashe and Zahs, 2010; Elder et al., 2010; Marchetti and Marie, 2011; Spires-Jones and Knafo, 2012; Peineau et al., 2018 or visit the Alzheimer forum at http://www.alzforum.org/res/com/tra/default.asp).

**Table 1:** The table summarizes relevant data relative to experimental Alzheimer’s disease (AD) models for which hippocampal electrophysiological analyses were performed.

Categories		Models	Species	Electrophysiological alteration				References
				BST	PPF	LTP	LTD	
In vitro models		Soluble Aβ oligomers treatment of hippocampal slices	Rat	–	–	↓ CA1	–	Lambert et al. (1998)
			Rat	NC		↓ DG	NC	Wang et al. (2002)
			Mouse	NC	NC	↓ CA1	↑ CA1	Shankar et al. (2008)
		APP over-expression in organotypic slices	Rat	↓	NC	↓ CA1	–	Kamenetz et al. (2003)
Transgenic animal models	APP-derived models	APP (K670N/M671L) (APP23)	Mouse	↓	NC	NC	–	Roder et al. (2003)
		APP (K670N/M671L) (Tg2576)	Mouse	NC	NC	↓ CA1	–	Chapman et al. (1999)
						↓ DG		
			Mouse	NC	NC	NC	↑ CA1	D’Amelio et al. (2011)
		APP (V717F) (APPind)	Mouse	↓	NC	NC	–	Hsia et al. (1999)
		APP (K670N/M671L)/APP (V717F)(J20)	Mouse	NC	NC	↓ CA1	NC	Nalbantoglu et al. (1997)
			Mouse	NC	↓	↓ DG	–	Palop et al. (2007)
		APP (K670N/M671L)/PS1 (P264L) (Tg2576/PS1)	Mouse	NC	NC	↓ CA1	↑ CA1	Lanté et al. (2015)
	PS1-derived models	PS1 (M146L)	Mouse	–	–	↑ CA1	–	Barrow et al. (2000)
		PS1 (WT)/PS1 (A246E)	Mouse	NC	NC	↑ CA1	–	Parent et al. (1999)
		PS1(A246E)	Mouse	–	NC	NC-CA1	–	Schneider et al. (2001)
		PS1 (L286V)	Mouse	NC	NC	↓ CA1	–	Auffret et al. (2009)
	APP/PS models	APP (K670N/M671L)/PS1 (M146L)	Mouse	↓	NC	↓ CA1		Trinchese et al. (2004)
		APP (KM670/671NL)/PS1 (L166P)	Mouse	NC	–	↓ CA1		Calella et al. (2010)
		APP (K670N/M671L)/PS1 (P264L)(Tg2576/PS1)	Mouse	NC	NC	↓ CA1	↑ CA1	Chang et al., (2006)
		APP (K670N/M671L)/PS1 (M146V)	Mouse	NC	–	↓ CA1	↓ CA1	Song et al. (2014)
		APP (K670N/M671L)/PS2 (N141I)	Mouse	NC	NC	↓ CA1	–	Richards et al.(2003)
				↑	↓	↓ DG	–	
	3xTg model	APP (K670N/M671L)/MAPT (P301L)/PS1 (M146V)	Mouse	↓	NC	↓ CA1	–	Oddo et al. (2003)
	5xFAD model	APP (K670N/M671L) /APP (I716V) /APP (V717I)/PS1 (M146L; L286V)	Mouse	↓	NC	↓ CA1	–	Kimura et al. (2009)

Models were grouped into in vitro models, APP-derived, PS1-derived, APP/PS1, 3xTg, and 5xTg models. Electrophysiological readouts include basal synaptic transmission (BST), paired pulse facilitation (PPF), long-term potentiation (LTP), and long-term depression (LTD). For each model, we also report the principal references to the electrophysiological studies.

#### APP-Derived Models

These mice models over-express the human APP, which is mutated in one or more sites. The single mutations introduced in the APP gene represent mutations associated with FAD, which are termed the Swedish (swe, K670N & M671L, Mullan et al., 1992), the Indiana (ind, V717F, Murrell et al., 1991; Hsia et al., 1999), the London (Ld, V171I, Goate et al., 1991), and the Arctic (E693G, Nilsberth et al., 2001) mutations. Other mouse models show a double mutation, such as the Swe mutation together with either the Indiana or Arctic mutation. These models manifest progressive Aβ accumulation and plaques similar to those found in humans. Aβ plaques observed in AD Tg mice brain appear structurally comparable to those discovered in the human brain; they start as diffuse plaques consisting mainly of Aβ 42 with a dense Aβ 42 core that contains Aβ 40 with many other non-Aβ components, among which are ubiquitin and synuclein (Yang et al., 2000). Moreover, these mice models show hyperphosphorylated tau and hippocampal-dependent memory deficits similar to human AD pathology, but do not show NFTs, cholinergic deficits, or neuronal death (Morrissette et al., 2009).

APP23 mice (Sturchler-Pierrat et al., 1997) display normal LTP in the hippocampus and prefrontal cortex at all ages tested (Roder et al., 2003). Tg2576 and the hAPPJ20 mouse models present an age-dependent reduction in LTP in the CA1 area (Nalbantoglu et al., 1997; Chapman et al., 1999; Fitzjohn et al., 2001; Balducci et al., 2011; D’Amelio et al., 2011) and in the dentate gyrus (Palop et al., 2007).

LTD has not been largely explored in APP-derived mouse models; only few studies have reported LTD measurement (D’Amelio et al., 2011; Cavallucci et al., 2013; Lanté et al., 2015). Both groups performed field recordings in hippocampal slices from Tg2576 mice and have shown enhanced NMDA receptor-dependent LTD starting already from 3 to 4 months of age.

In addition, a recent study described alteration of mGlu-dependent LTD in 4-month-old Tg2576 mice, elicited by perfusion of the group I mGlu agonist DHPG (Mango and Nisticò, 2018).

#### PS1-Derived Models

These models over-express the human presenilin gene (PS1) encoding an FAD mutation. Being part of the secretase complex, this gene is involved in the APP proteolysis. Presenilin variants do not produce neuropathology, but potentiate plaque deposition in APP transgenic mice. Electrophysiological studies have been performed on M146L, A246E, and L286V mutants (Parent et al., 1999; Barrow et al., 2000; Schneider et al., 2001; Auffret et al., 2009). A mouse model was also generated harboring the PS1ÆE9 FAD mutation, which results in a functional and non-cleavable modification of PS1 (Zaman et al., 2000). One knock-in mouse was engineered in which mouse PS1 was exchanged by its mutant M146V (Sun et al., 2005). These presenilin FAD mutants consistently exhibit an age-dependent increase of Aβ42 with minor effect on Aβ40; however, they do show amyloid plaques, tau pathology, cholinergic alterations, or neurodegeneration and present only a mild cognitive deficit (Games et al., 2006). Most studies report that young adult (up to 6 months) transgenic mice over-expressing PS1M146L, PS1M146V, PS1A246E, PS1ΔE9, or PS1L286V display enhanced CA1-LTP elicited using different conditioning protocols (Schneider et al., 2001; Oddo et al., 2003; Dewachter et al., 2008; Auffret et al., 2009). So far, no study described LTD in these mouse models.

#### APP/PS1 Models

To accelerate the brain Aβ accumulation and plaque aggregation, researchers crossed APP- and PS1-derived animal models. Most electrophysiological studies focused on double transgenic models harboring the human APPswe transgene together with the PS transgene.

APP/PS1 double mutant mice develop rapid and extensive Aβ plaque accumulation, tau pathology, and cognitive defects, even though they lack cholinergic deficits, neuronal loss, and NFTs (Morrissette et al., 2009).

Most studies report a reduction of LTP in APPswe/PS1M146L (Gong et al., 2004; Trinchese et al., 2004, Trinchese et al., 2008), APPswe/PS1P246L (Chang et al., 2006), APPswe/PS1L166P (Calella et al., 2010), and APPswe/PS2N141I (Richards et al., 2003) mice. Using a standard LFS protocol, Chang and coauthors (2006) report a linear decline in CA1-LTD expression between 9 and 20 months of age in the APPswe/PS1P246L model. Moreover, loss of LTD was described in the APPswe/PS1M146V mouse model (Song et al., 2014). Also, a recent study has shown mGlu receptor-LTD impairment in the APPswe/PSEN1/ΔE9 (Yang et al., 2016), whereas no alteration in the basal transmission was found in this model (Volianskis et al., 2010).

#### 3xTg Model

The 3xTg model over-expressing human APPswe and tau MAPTP301L and encoding a knock-in of PS1M146V was generated for the first time by Oddo et al. (2003). These mice display both Aβ aggregates and NFT and show hippocampal-dependent memory decline during aging. Also, they show cholinergic alterations and neuronal loss in the cortex (Oddo et al., 2003; Perez et al., 2010). This model is advantageous since it presents a significant intracellular Aβ deposition before the occurrence of extracellular plaques, which become evident around 12 months of age, and notably, it develops NFTs comparable to humans.

Concerning the functional aspects, 6-month-old 3xTg mice display impairment of LTP (Oddo et al., 2003), which correlates with intracellular Aβ well before plaque and tangle pathology. Recently, other triple transgenic mice have been generated harboring APP, PS2, and tau mutations (Rhein et al., 2009; Grueninger et al., 2010) but electrophysiological investigations have not been performed so far.

#### 5xFAD Model

5xFAD mice (Tg6799 line) harbor three APP and PS2 (M146V and L286V) mutations that are causally related to FAD (Oakley et al., 2006). They exemplify one of the most early-onset mouse models with robust amyloid pathology (Oakley et al., 2006; Ohno et al., 2006, Ohno et al., 2007). Indeed, 5xFAD mice start developing amyloid deposition already from 2 months of age and show early hippocampal dysfunction, as evidenced by reduced basal synaptic transmission and LTP (Kimura and Ohno, 2009; Crouzin et al., 2013). This mouse model exhibits a strong pathology: at 1.5 months of age mice already express intracellular Aβ42, which massively progresses at 2 months of age with extracellular Aβ accumulation, senile plaques, and lack of specific neuronal populations. Cognitive impairment is reported at 4–6 months of age (Oakley et al., 2006). LTD has not yet been investigated in this mouse model.

## Clinical Relevance of LTP/LTD

Many of the mechanisms underlying LTP and LTD in the rodent hippocampal slice preparation are shared also in hippocampal tissue from patients undergoing surgery for intractable temporal lobe epilepsy. Indeed, LTP is induced in the temporal lobe and in the dentate gyrus in humans using similar protocols of stimulation (Chen et al., 1996; Beck et al., 2000) and is modulated by the different pharmacological approaches just as in non-clinical models. These studies further support the notion that also the human brain manifests LTP- and LTD-like events even though linking synaptic plasticity to human learning and memory remains a challenge (Bliss and Cooke, 2011). It also should be kept in mind that the human tissue investigated in electrophysiological studies is found in a pathological state, deriving from patients with an epileptic focus in the temporal lobe.

Notably, LTP- and LTD-like events are nowadays exploited in humans for therapeutic purposes. These plastic changes can be induced through several noninvasive techniques such as repetitive transcranial magnetic stimulation (rTMS) and transcranial direct current stimulation (tDCS) and are used for the treatment of a variety of neurological and psychiatric conditions, such as epilepsy, drug addiction, depression, Parkinson disease, neuropathic pain, tinnitus, and stroke (Schulz et al., 2013).

We can therefore hypothesize that stimulation of neuroplasticity in the early stages of AD, through pharmacological and noninvasive approaches, can attenuate disease progression. Even though numerous therapeutic interventions reverse synaptic alterations and improve behavior in the non-clinical AD models, so far, there has been no successful translation into disease-modifying compounds in humans (Nisticò et al., 2012).

## Predictive Value of AD Models

Considering the recent clinical trial failures in AD, there has been considerable discussion as to whether results obtained from non-clinical models are predictive or simply misleading. There are numerous reasons why non-clinical studies may have failed to predict clinical trial outcome. One of the main issues it that Tg mice carry FAD mutations, accounting for only 5–10% of all AD cases, while the vast majority of AD cases are sporadic (sAD). As a consequence, these models have a low face and predictive validity for the sporadic form of AD. Moreover, transgenic models normally overexpress APP with consequent overproduction several APP fragments. Of note, a knock-in mice with a phenotype more similar to human was recently generated (Saito et al., 2014). Another limitation is that each mouse model develops only specific characteristics of AD (i.e. Aβ *vs*. tau pahology) and does not recapitulate the complexity of the human disease. It has also to be taken into account whether animal models display a similar spatiotemporal profile of disease progression when compared to AD patients. For example, cognitive deficits usually precede plaque load in mice, whereas the opposite occurs in patients. An important issue to be considered is that the majority of AD models lack neuronal cell death, while a substantial neurodegeneration is observed in the human AD brain.

It can be argued that the various non-clinical models typify specific disease-related targets and pathways, a potential advantage for testing candidate molecules on selected targets involved in AD pathogenesis. Indeed, this target-driven approach in non-clinical models has been translated over the years into numerous clinical studies (Nisticò et al., 2012).

In addition to intrinsic limitations of animal models, experimental bias is another crucial factor. For example, gender- and litter-dependent differences, variability in transgene expression, and the different genetic background among models and even between active treatment and placebo groups should all be considered in translation. Also, diversities in brain anatomy, neuronal physiology, metabolism, and disease susceptibility play a central role. Moreover, given the complex dynamics of drug-target interactions, *in vivo* studies in non-clinical models should include a complete pharmacokinetics/pharmacodynamics profile in order to ensure that the dose range and timing are specific to the target (e.g. based on receptor occupancy and/or correlative biomarker data). On the other hand, the therapeutic window and the off-target effects that may lead to adverse effects should also be identified especially for behavioral experiments.

To improve clinical translation, non-clinical endpoints need to be accurately selected and should employ a combination of disease-relevant approaches such as integrated neurophysiological/behavioral paradigms. Electrophysiological techniques record neural activity from large neural populations down to single cells and are ideal to measure synaptic plasticity, as well as firing activity and neural oscillations. A limitation is that this approach does not address spatiotemporal information and is not suitable for noninvasively detecting activity from deep brain regions. In contrast, functional neuroimaging provides a noninvasive spatiotemporal readout of changes in brain function, making it an invaluable tool for most clinical studies. Unfortunately, the use functional imaging has been limited in non-clinical setting due to the restricted applicability to animal models and the relatively high cost.

## Conclusions

We have previously hypothesized that alterations in normal synaptic function are not only a key feature but also a leading cause of disease (Nisticò and Collingridge, 2012). In this respect, LTP and LTD can serve as synapse survival and death signals, respectively. Thus, conditions that promote LTD, i.e. following excessive Aβ load in the early-onset forms of disease, can lead to loss of synapses. On the other hand, promoting LTP, which is known to inhibit LTD (Peineau et al., 2007), can represent a protective mechanism to preserve synaptic plasticity and brain connectivity.

It seems important to investigate the molecular mechanisms that influence plasticity in the human brain and to determine whether its vulnerability to aging and neurodegeneration can be modified by pharmacological intervention. Considering that AD is a complex disease affecting multiple signaling pathways, therapeutic strategies should not be directed to a single target rather to a combination of targets. To ensure a successful outcome, therapy should start at an early stage of disease. In addition, highly sensitive and specific biomarkers should identify susceptible individuals at the onset of disease (Hampel et al., 2014).

Generally, the predictive value of non-clinical models in the drug discovery process has been largely debated independently of the therapeutic area (McGonigle and Ruggeri, 2014; Mullane and Williams, 2019). Accordingly, several compounds showing robust efficacy in experimental models of AD have failed so far in clinical trials. Once a lead compound is selected, selection of non-clinical endpoints through integrated approaches should reflect the clinical endpoints in phase I studies. Correct design of non-clinical studies can be a long, complex, and expensive process that may slow down the course of drug development (Mohs and Greig, 2017); nonetheless, the probability of successful approval and hence time saving and return on investment is certainly increased.

## Author Contributions

DM, AS and GC prepared the manuscript and reviewed the drafts, MF and MC reviewed the drafts, RN conceived the idea, prepared the manuscript and reviewed the drafts. All authors contributed to the writing and final approval of the manuscript.

## Funding

This work was supported by Fondazione Turano.

## Conflict of Interest Statement

The authors declare that the research was conducted in the absence of any commercial or financial relationships that could be construed as a potential conflict of interest.

## Abbreviations

Aβ, amyloid β protein; Aβo, β-amyloid protein oligomers aggregates; AD, Alzheimer’s disease; AMPAR, L-α-amino-3-hydroxy-5-methyl-4-isoxazole propionate receptor; APP, amyloid precursor protein; BST, basal synaptic transmission; E-LTP, early-LTP; FAD, familiar Alzheimer’s disease; LFS, low frequency stimulation; LTD, long-term depression; LTP, long-term potentiation; L-LTP, late- LTP; mGlu, metabotropic glutamate receptor; NFT; neurofibrillary tangles; PPF, paired pulse facilitation; Tg, transgenic; WT PS1, wild-type human PS1; NA, not assessed; rTMS, repetitive transcranial magnetic stimulation; sAD, sporadic Alzheimer’s disease; tDCS, transcranial direct current stimulation.
